# Sarcoma of Lower Jaw—Varicocele—Rarefying Ostitis of Tibia—Deformity of Lower Extremity—Angeio-Sarcoma of Testis

**Published:** 1893-07

**Authors:** Charles M'Burney

**Affiliations:** Professor of Surgery at the College of Physicians and Surgeons, New York; Visiting Surgeon to Rossevelt Hospital, etc.


AMERICAN JOURNAL

—OF—

DENTAL SCIENCE.

Vol. xxvii. Baltimore, July, 1893. No. 3.

ARTICLE I.

### SARCOMA OF LOWER JAW—VARICOCELE—RAREFYING OSTITIS OF TIBIA—DEFORMITY OF LOWER EXTREMITY—ANGEIO-SARCOMA OF TESTIS.

BY CHARLES M'BURNEY, M. D.

Professor of Surgery at the College of Physicians and Surgeons, New York; Visiting Surgeon to Rossevelt Hospital, etc.

The first case that I show you is one of very great interest from several points of view. The patient is a young woman, twenty-two years of age, who came under my observation some three years ago, complaining of the existence of a considerable tumor on the lower jaw. She gave a history of having noticed some enlargement two years before, due, as she says, to a blow received there some twenty months previously.

The tumor, when I first saw it, was about as large as a pigeon's egg, rather elongated in form, occupying the region to the right of the symphysis and two inches backward. The teeth are perfect and undislodged. The growth is very hard to the touch, somewhat nodulated, and there is no involvement of the mucous membrane, nor attachment to the soft parts over it.

When I first saw the patient I said that I considered this growth was an osteo-sarcoma of the lower jaw. She remained away for a little while and came back for a second examination. On re-examination, owing to the outline and consistence of the tumor, which felt very much like a cartilaginous or bony growth, I came to the conclusion that it was an osteomia of a non-malignant character. I put the patient under ether and made an incision along the lower jaw with a view of removing it, if it was an osteoma. On removal of the surface of the tumor I became suspicious of its character. It looked more like a sarcomatous growth than anything else; I had the tissue that was removed examined, closed the wound, and the report came from the laboratory that the growth was a pseudo, small-celled sarcoma.

The treatment of the case resolves itself into a complete extirpation of the disease itself and of the tissue involved, *viz:* the rest of the jaw on that side of the face. If the disease were treated by a simple removal of only the affected portion, there would be a liability to a recurrence in the remaining part. Moreover, leaving the ramus of the jaw behind will not prove of any value to the patient. The portion which is left will become tilted by the temporal muscle and external pterygoid, causing interference with the use of the other side of the jaw.

What I propose to do is to completely extirpate the disease by the removal of one-half of the lower jaw. I want to call your attention particularly to the condition in which a patient is left, who has had one half of the inferior maxilla removed. It is not a difficult operation and the indications are perfectly plain. If you look at your text-books you will find no reference made to this condition. The patient is spoken of as free from disease; but any of you who have seen such a patient after removal of the lower jaw, will notice the position of the face. The unopposed muscles of the opposite side throw the remaining portion of the jaw directly into the mouth, so that the patient has his mouth practically filled with the unremoved half of the jaw. He is able to get along without trouble, if there is no recurrence, but he is a very pitiable object to look upon. I attempted some time ago to construct a mechanical device that would overcome this difficulty following the operation. It seemed to me that if the proper ingenuity could be brought to bear upon the subject, if the uninjured part of the lower jaw could be held in position till the wound healed, the difficulty spoken of could, to a large extent, be remedied. I therefore asked a dentist of this city to make such a contrivance for me. He succeeded in making an interdental splint which was put on the upper jaw and the lower half of the jaw that was left after the operation. This plan succeeded very well, indeed. The splint is a simple one of the same kind as has been used for holding together fragments of a broken jaw. It is made of rubber, by taking first a plaster cast of the patient's jaws, so that the spaces for the teeth may be accurately adapted to the splint, and then the splint is made on this plaster cast. The splint fits absolutely everyway. When I get through with the operation, I expect to slide this splint into position so as to keep the remaining fragment of jaw in proper position with the other and keep it there till the wound is healed.

The operative technique in this case is a very simple one. I shall make an incision starting from the median line and running towards the angle of the jaw. This will be supplemented by a vertical one extending from the angle of the jaw upwards along the posterior border of the ramus as far as the middle of the lobe of the ear. I shall dissect the soft parts away from the outer surface of the bone and the muscles, etc., from the inner surface as far as the mucous membrane, keeping well away from the tumor.

The steps of the procedure, as detailed here, were carried out by the operator, the mucous membrane being cut through. The left bicuspid tooth was then extracted and the inferior maxilla broken with a rongeur at this point. The pterygoid muscles were next divided and the bone removed by disarticulation. The interdental splint was adjusted in position before the patient left the operating table, so as to illustrate its applicability to the students present. It was then removed until the patient recovered from the anæsthetic, when it will be reintroduced and will be kept in position, except during the necessary irrigation, until the patient shall be discharged cured. The usual dressings were applied.

The next patient is a young man who has a varicocele, without any complication, or any known cause to direct your attention to the fact that he is suffering from such an affection. I have had a good many patients who come under my care because they want to pass through some physical examination for West Point or a similar institution, where they are rejected because of this trouble.

This man wishes to pass such an examination and I have asked him if he has any of the symptoms mentioned in the books as due to varicocele, but he says he has none whatever. I think, myself, varicocele is of comparatively little importance in the large majority of cases, so far as the health of the individual is concerned, and yet one does find a certain number of cases where disturbances take place, which disappear after the cure of the varicocele. In a certain number of cases the testes will be reduced to the size of a large bean, without any other cause for such a reduction. In this case the testes are practically alike in size. When a patient complains of discomfort from a varicocele, pains in the groin or back or other morbid manifestations, I think under such circumstances an operation is well indicated.

When you come to read over the list of palliative treatment, you begin to be really unhappy. It consists in wearing a suspensory bandage, summer and winter, bathing the scrotum in cold water, keeping the bowels open, avoiding the use of spirituous liquors absolutely, non-indulgence in sexual intercourse, and leading a kind of life a great many people would object to. Such a treatment as this, in view of modern surgical methods, is really absurd, inasmuch as such a surgical operation is usually extremely successful and the results very satisfactory.

The subcutaneous operation is, I think, largely passing out of favor, because the open method is so simple and safe. Ten years ago every one was devoted to the subcutaneous ligation of the large veins, and in every part of the world it was being done. In the majority of cases this subcutaneous method does cure, though I have seen patients in whom this procedure had not been successful. I do not like the subcutaneous operation because it has no particular advantage. In the first place it may fail to cure the patient, and the patient in consequence of the swelling of the testis, has to remain as long a time in bed as after the other method, and if there is a septic condition of the skin, the passage of sutures may give rise to abscesses, leading to a protracted illness. The open operation which is to be preferred, is very simple and very safe, as I have already said. If the testicle is seized below, and the penis put upon the stretch so as to tighten the lax skin, it is easy to make a straight incision through the different layers of the tissue until the veins are clearly seen. When you have removed the veins, you have radically cured the varicocele.

The next patient is a boy, thirteen years of age, who was operated upon for a rarefying ostitis involving the whole length of the tibia; and also for a deformity at the ankle joint, which was anchylosed in a very unseemly position. The boy is a tuberculous subject, and had many osseous lesions which have now healed.

An amputation was done above the knee-joint, and the temperature has been below 100° F. since the operation. The wound is now, as you see, in a very healthy condition and primary union has taken place throughout its entire length.

The next patient operated upon presented a periosteal sarcoma of the tibia. The disease was a recurrent one and had been operated upon two years previously. The only thing to be considered in this connection was amputation at the next joint above, *viz:* the knee-joint. I did the operation according to Stephen Smith's plan, but the result has not been so satisfactory. The patient went on perfectly well for four or five days, when he developed some fever, and yesterday I found on taking off the dressings that while the wound was perfectly aseptic, a considerable accumulation of synovial fluid had taken place in the pouch of the patella, which is one of the things that we fear in this amputation, and had produced enough distention of the pouch to give the patient a considerable amount of fever.

I administered chloroform, made a free incision into the pouch above the patella, and evacuated a quantity of pure synovial fluid. I was sorry I did not do this at the time of operation, but I think it will now bring the patient into a proper condition.

I washed out the sac with bichloride solution, and now under free drainage, the case will go on to complete recovery.

The next case is a man who has a perfectly useless knee and a leg bent at right angles to the axis of the limb. He is an Italian, twenty four years of age. Some twelve years ago he was shot through the lower end of the femur. The bullet entered on the outside of the thigh and escaped on the inside, either penetrating the femur or going directly behind it. As the man is an Italian and not able to speak the English language, I was unable to obtain a very clear history of the case from him. However, it is plain that he received this injury, and also that he had a long suppurative process following the injury which confined him in bed for a considerable length of time. He fortunately got well and we do not know whether there was a suppurative disease of the knee-joint or not, or suppuration of the extra-capsular tissues behind the joint.

This man, however, furnishes us with a good illustration of the mistakes that is often made, and is being made constantly by medical practitioners in treating cases of inflammation of the joints, and particularly of the rheumatic or gouty kind, which is to allow the patient to assume any position he is comfortable in, and remain in that position until a cure of the disease is brought about. That is the way in which this man has been treated; he has been allowed to remain with the knee flexed, and when he got over the suppurative process the condition you now see resulted, the leg being bent at right angles to the femur, and the man is now walking on his toes.

Anchylosis is not complete here, but there is a great deal of displacement of the head of the tibia back on the condyles of the femur. One has to remember that after remaining for so long a time in this position the tissues have shortened, and any attempt to straighten the limb by forcible extension is very apt to result in rupture of the artery, vein or other important vessels or nerves. Such accidents as this have occurred in the hands of surgeons who have attempted this manœuvre.

The question is what ought the surgeon do in this case to produce a useful limb ? What I shall do for this man is to bring the leg out to a straight line with the femur by operative procedure, and obtain firm anchylosis of the leg in a proper position. The only way that can be done is by excision of the joint. If we can excise a sufficient area of tissue of a triangular shape, with the apex directed backwards, straighten the leg and then bring the bony parts together, and get good bony union, he will have a strong, useful limb, though with a certain amount of shortening. He will be able then to walk upright, and with a thick sole, he will be able to walk with absolute ease and comfort.

The operation itself is an extremely satisfactory one in all of these cases of deformity, and is an especially safe one since the introduction of antisepsis. When I was a student we were told that anything was to be preferred to an excision of the knee-joint, and such a rule was laid down by all the text books. Every surgeon at that time preferred to do an operation at the thigh than at the knee. Now we hardly ever lose a patient from excision of the knee-joint, and primary union generally results with an extremely useful limb.

After I have removed a sufficient amount of the femur and tibia, so as to bring those bones in apposition, I shall make use of strong catgut sutures for bringing the bony surfaces together. Nails and wires have both disadvantages in this respect, in that they crush a good deal of bone in going through, and they act as foreign bodies. They are liable also to cause ulceration afterwards and not infrequently they give rise to very obstinate sinuses. The real fact seems to be that it is necessary to hold bones together only until a permanent dressing may be applied with safety. Catgut is quite strong enough for that purpose, and also it takes care of itself and there is nothing to be removed afterwards. The dressing which I shall apply I will not disturb for three weeks, when I will show you the patient again.

The next patient gives a history of an enlargement on the right side of the scrotum since last October. It has been painless in its development, is quite smooth and has some elasticity. He was examined before he came here, and his case was diagnosticated as one of hydrocele. A needle was inserted and a quantity of clear, serous fluid was withdrawn. He came here, and on examination we find he has a solid tumor of the testis of considerable size. A section was removed and I have had it examined under the microscope, and it proves to be a case of angio-sarcoma of the testis.

The tumor is constantly growing; it seems to be at present confined to the testis, and on examination of the abdomen I have not been able to discover any enlargement of the lumbar glands. The cord is free from involvement; still we know that the prognosis in such a case as this is extremely bad, and yet the individual is given a chance of cure by radical removal. There are few cases in which after the disease has reached this point the patient has gone on to complete recovery. It is likely that in a few years secondary deposits will appear in the abdominal cavity.

What I propose to do here for the immediate relief of the pain, is to dissect the tumor from its surroundings, and if it is not attached to the skin at any point, enucleate it from the scrotum, and then follow it up, making a section of the cord at a high level.—*International Journal of Surgery.*

---

ARTICLE II.

